# Diagnostic Accuracy of Unattended Automated Office Blood Pressure Measurement in Screening for Hypertension in Kenya

**DOI:** 10.1161/HYPERTENSIONAHA.119.13574

**Published:** 2019-10-28

**Authors:** Anthony O. Etyang, Antipa Sigilai, Emily Odipo, Robinson Oyando, Gerald Ong’ayo, Lawrence Muthami, Kenneth Munge, Fredrick Kirui, Jane Mbui, Zipporah Bukania, Judy Mwai, Andrew Obala, Edwine Barasa

**Affiliations:** 1 From the Epidemiology and Demography Department, KEMRI-Wellcome Trust Research Programme (A.O.E., A.S., E.O., G.O.); 2 Health Economics Research Unit, KEMRI Wellcome Trust Research Programme, Nairobi, Kenya (R.O., K.M., E.B.); 3 Centre for Public Health Research (L.M., Z.B., J.M.), Kenya Medical Research Institute, Nairobi; 4 Centre for Clinical Research (F.K., J.M.), Kenya Medical Research Institute, Nairobi; 5 Moi University, Eldoret, Kenya (A.O.).

**Keywords:** adult, blood pressure, humans, hypertension, Kenya

## Abstract

Despite increasing adoption of unattended automated office blood pressure (uAOBP) measurement for determining clinic blood pressure (BP), its diagnostic performance in screening for hypertension in low-income settings has not been determined. We determined the validity of uAOBP in screening for hypertension, using 24-hour ambulatory BP monitoring as the reference standard. We studied a random population sample of 982 Kenyan adults; mean age, 42 years; 60% women; 2% with diabetes mellitus; none taking antihypertensive medications. We calculated sensitivity using 3 different screen positivity cutoffs (≥130/80, ≥135/85, and ≥140/90 mm Hg) and other measures of validity/agreement. Mean 24-hour ambulatory BP monitoring systolic BP was similar to mean uAOBP systolic BP (mean difference, 0.6 mm Hg; 95% CI, −0.6 to 1.9), but the 95% limits of agreement were wide (−39 to 40 mm Hg). Overall discriminatory accuracy of uAOBP was the same (area under receiver operating characteristic curves, 0.66–0.68; 95% CI range, 0.64–0.71) irrespective of uAOBP cutoffs used. Sensitivity of uAOBP displayed an inverse association (*P*<0.001) with the cutoff selected, progressively decreasing from 67% (95% CI, 62–72) when using a cutoff of ≥130/80 mm Hg to 55% (95% CI, 49–60) at ≥135/85 mm Hg to 44% (95% CI, 39–49) at ≥140/90 mm Hg. Diagnostic performance was significantly better (*P*<0.001) in overweight and obese individuals (body mass index, >25 kg/m^2^). No differences in results were present in other subanalyses. uAOBP misclassifies significant proportions of individuals undergoing screening for hypertension in Kenya. Additional studies on how to improve screening strategies in this setting are needed.

## Abstract 2

Supplemental Digital Content is available in the text.

**See Editorial, pp 1294–1296**

Methods used to measure blood pressure (BP) worldwide in both nonresearch and research settings tend to be those that have been used in major cardiovascular outcome trials,^1^ nearly all of which have been conducted in high-income countries. Unattended automated office BP measurement (uAOBP) in which BP is measured using an oscillometric device in a quiet room with no observer present^2^ was the primary procedure utilized in the landmark SPRINT (Systolic Blood Pressure Intervention Trial).^3^ Advantages of uAOBP are that it minimizes the proportion of individuals with the white coat effect (false positives),^4^ and the geographic location of measurement does not appear to influence the results as long as the procedure is performed with the patient alone in a quiet room.^2^ In addition, several studies^5–7^ have demonstrated that uAOBP predicts target organ damage, as well as ambulatory BP (ABP) measurement, which is considered the reference standard for BP measurement.^8^

Despite increasing adoption of uAOBP,^9–11^ no studies examining its utility as a screening tool for hypertension have been conducted to date, leading to some confusion on how to incorporate uAOBP in the diagnosis of hypertension.^12^ While there are data comparing uAOBP to ABP monitoring (ABPM), these have been conducted among high cardiovascular risk individuals, the majority of whom were already on treatment for hypertension.^5,6,13–18^ Participants in these studies, which were additionally limited by their small sample size (median sample size, 226^18^), were generally older (age, ≥60 years)^5,6,13–18^ than patients undergoing screening for hypertension in Africa.^19,20^ Another limitation of previous studies is the fact that they have had small numbers of individuals of African descent, and have reported comparisons of uAOBP limited to awake ABP measures, and yet 24-hour ABPM is a better predictor of cardiovascular outcomes.^4^ In addition, uAOBP cannot detect nocturnal nondipping and masked hypertension (false negatives on office measurement), conditions that confer high cardiovascular risk and appear to be highly prevalent among individuals of African descent.^21–23^

These and other limitations^24^ of previous studies mean that their findings may not be applicable to other populations that are likely to adopt uAOBP as the primary method of screening for hypertension. We determined the validity and levels of agreement between uAOBP and ABPM measurements in a sample of adults drawn from the general population at 3 sites across Kenya.

## Methods

### Data and Material Availability

The full study data contained in this article are available by writing to dgc@kemri-wellcome.org. The data can only be used for purposes consistent with consent given by study participants.

The study was conducted at 3 geographically distinct sites in Kenya: Kilifi, located along the Indian Ocean coast; Kirinyaga, in the central highlands; and Webuye, in Western Kenya (Figure S1 in the online-only Data Supplement). In Kilifi and Webuye, we selected an age-stratified random sample of adults (age, ≥18 years) to participate in the study drawn from population lists that are updated regularly as part of the Kilifi and Webuye Health and Demographic Surveillance Systems.^25,26^ In Kirinyaga, where there is no existing Health and Demographic Surveillance System, we first conducted an enumeration exercise using the same methods applied in Webuye and Kilifi, where we listed all of the individuals living in 2 administrative locations, before randomly selecting an age-stratified sample to participate in the study.

The study was conducted between June 2017 and December 2018. Trained staff visited all potential participants at their homes and requested them to come to the study clinics to undergo study procedures. Participants received reimbursement for travel costs and lost wages. Participants who reported that they were pregnant or were suffering from a chronic illness, for example, chronic kidney disease, were excluded.

uAOBP measurement was performed at study clinics using the Omron HEM 907 device (Omron, Kyoto, Japan),^27^ which was also used in SPRINT.^3^ The measurement procedure fully complied with the most stringent unattended BP measurement conditions used in SPRINT.^28^ Study staff attached appropriately sized cuffs to the nondominant arm of the participant and programmed the device to automatically inflate after 5 minutes. Study staff then left the room, and after 5 minutes of quiet rest, the sphygmomanometer inflated 3× at 1-minute intervals. The average of the 3 measurements was recorded as the participants’ clinic BP.

After completing the uAOBP measurement, participants were immediately fitted with an ABPM device. We used Arteriograph 24 (TensioMed, Ltd, Budapest, Hungary).^29^ An appropriately sized cuff was fitted on the nondominant arm and the device programmed to inflate every 20 minutes during the day and every 40 minutes at night. Participants were advised not to remove the devices at any time and to continue with their normal activities during the course of measurement and return to the clinic after 24 hours for removal of the monitor. All devices used in the study had undergone calibration by their respective manufacturers in the year preceding the study. The same staff conducted the uAOBP and ABPM measurements and were not blinded to any of the results.

### Statistical Methods

We followed the Standards for Reporting Diagnostic Accuracy guidelines in reporting results and recommendations from the TRUE (inTernational consoRtium for qUality resEarch on dietary sodium/salt) on standards for reporting on hypertension studies.^30,31^ The sample size was designed to provide overall sensitivity with a precision of ±5% assuming a crude prevalence of true hypertension of ≥25% and φ (the probability of generating enough cases for the study) specified at 0.90.^32^ ABPM data were included in the analyses if they met the International Database of Ambulatory Blood Pressure in relation to cardiovascular outcomes criteria for completeness. These criteria require a minimum of 10 readings during the daytime (1000–2000 hours) and a minimum of 5 nighttime (0000–0600 hours) readings.^33^ The same time periods were used to determine average daytime and nighttime BPs. Twenty-four–hour BP averages were calculated using all available readings, applying time weighting to account for the different frequency of daytime and nighttime measurements.^34^

Validity measures were computed using the combination of uAOBP (screening) and 24-hour ABPM (confirmatory) results. We defined screen-positive participants using 3 different cutoffs of mean uAOBP systolic BP (SBP)/diastolic BP: ≥130/80, ≥135/85, and ≥140/90 mm Hg reflecting the different international criteria that exist for diagnosing individuals as hypertensive based on conventional office and uAOBP measurements.^8,35^ We defined confirmed hypertensives as those whose ABPM results met European Society of Hypertension–defined threshold for hypertension on 24-hour ABPM: this definition diagnoses hypertension on ABPM based on an individual having a 24-hour BP average ≥130/80 mm Hg. Ambulatory awake hypertension was defined as daytime ABP ≥135/85 mm Hg.^8^ Nocturnal hypertension was defined as nighttime ABP ≥120/70 mm Hg.^8^

In the primary analysis, we excluded participants (n=65) who reported that they were on antihypertensive medication. We categorized study participants using the combination of uAOBP measurements and ABPM into 4 groups: sustained hypertensive (screen-positive on uAOBP and confirmed hypertensive on ABPM), white coat hypertensive (screen-positive on uAOBP but not confirmed hypertensive on ABPM), masked hypertensive (screen-negative on uAOBP but confirmed hypertensive on ABPM), or normotensive (screen-negative on uAOBP and not confirmed hypertensive on ABPM). The 4 categories were used to compute age-stratified measures of validity of uAOBP with ABPM as the reference standard. Sensitivity, specificity, positive and negative predictive values, and positive and negative likelihood ratios were calculated as simple proportions with corresponding 95% CIs. Discrimination as a measure of overall diagnostic accuracy was assessed using area under the receiver operating curves.^36^ The χ^2^ and McNemar tests, where appropriate, were used to assess statistical significance of differences among proportions. Student *t* test was used in assessing differences between continuous variables. All *P* values involved hypothesis tests against a 2-sided alternative and were considered significant when *P* was <0.05. We performed sensitivity analyses to examine the effect of including data from participants (n=229) whose ABPM measurements did not meet quality criteria. In addition, we examined the effect of including participants who reported having taken antihypertensive medication. Stratified analyses were done according to sex, age group, study site, and body mass index (BMI) category. We in addition conducted multivariable linear regression analysis to test whether age, sex, study site, BMI, diabetes mellitus, or current smoking status influenced the difference between uAOBP and ABPM measurements.

We measured levels of agreement between ABPM-derived measures and uAOBP using Bland-Altman plots^37^ and determined the correlation between the means and differences in these plots using Pitman test.

All analyses were conducted using Stata, version 15, software (College Station, TX).

The Ethical Review Committee of the Kenya Medical Research Institute approved the study. Written informed consent was obtained from all study participants.

## Results

**Figure 1.**
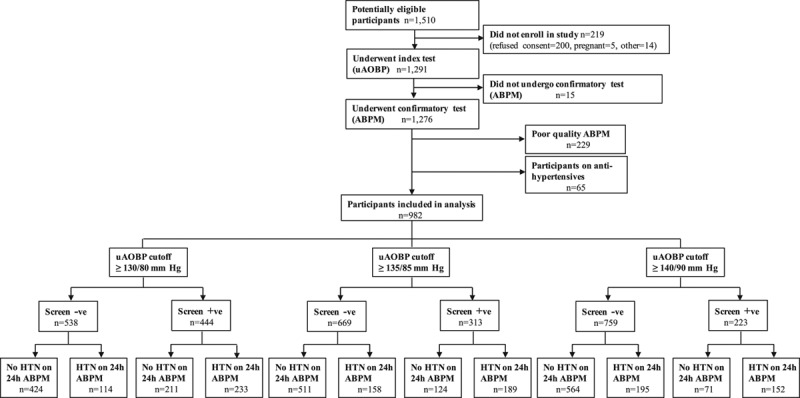
Study flowchart. Hypertension (HTN) on ambulatory blood pressure monitoring (ABPM) was defined as 24-h ABPM value ≥130/80 mm Hg. uAOBP indicates unattended automated office blood pressure.

We recruited 1291 (85%) of 1510 potentially eligible individuals into the study (Figure 1; Figures S1 through S3). Characteristics of study participants are displayed in Table 1 and Table S1. Data from 219 participants were excluded from the analyses because they did not meet ABPM quality criteria. There were no significant differences between participants who were included (n=982) and those who were excluded from the analysis (Table 1). Diabetes mellitus was present in 2% of study participants. After adjusting for age, sex, and BMI, there were no significant differences in any BP measure between any of the study sites.

**Table 1.**
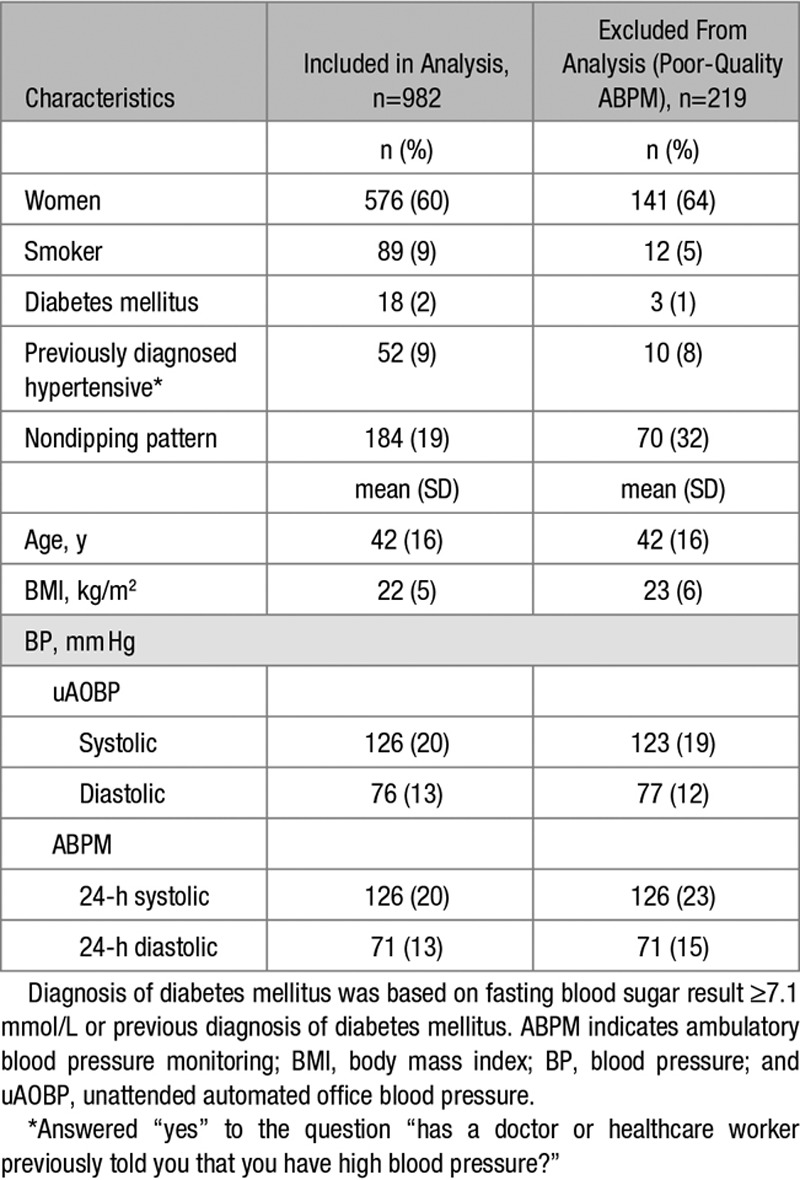
Characteristics of Study Participants

### Level of Agreement Between ABPM and uAOBP Measurements

**Figure 2.**
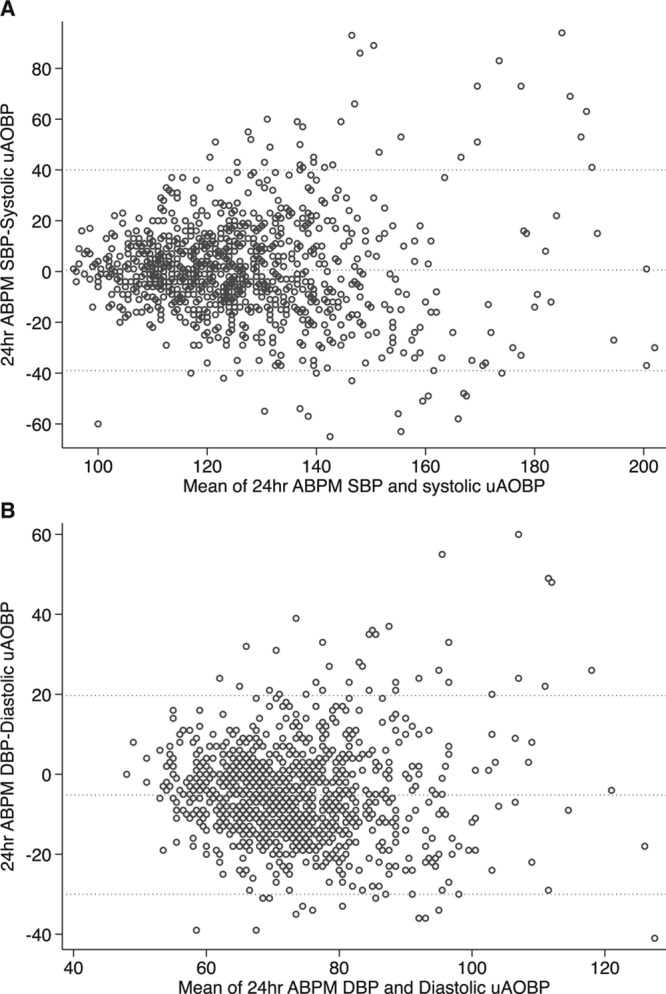
Bland-Altman plots showing levels of agreement between unattended automated office blood pressure (uAOBP) and ambulatory blood pressure monitoring (ABPM) measurements. **A**, Systolic; (**B**) diastolic. The correlation coefficients (Pitman test) between the difference and mean were r=−0.021, *P*=0.516 for systolic measurements and r=0.002, *P*=0.945 for diastolic measurements indicating no systematic proportional trend. Dashed horizontal lines indicate 95% limits of agreement. DBP indicates diastolic blood pressure; and SBP, systolic blood pressure.

A Bland-Altman plot revealed poor agreement between ABPM and uAOBP measures with wide limits of agreement (−39 to 40 mm Hg; Figure 2). This was despite there being overall no difference between mean 24-hour SBP on ABPM and mean SBP on uAOBP (Table 2). Mean daytime (awake) SBP on ABPM was significantly higher than mean SBP on uAOBP (+6.9 mm Hg; CI, 5.6–8.1). There was a statistically significant correlation (*P*<0.001) between uAOBP and ABPM measures, with coefficients ranging from 0.48 to 0.51 for systolic measures (Table 2) and 0.51 to 0.55 for diastolic comparisons (Table S2).

**Table 2.**
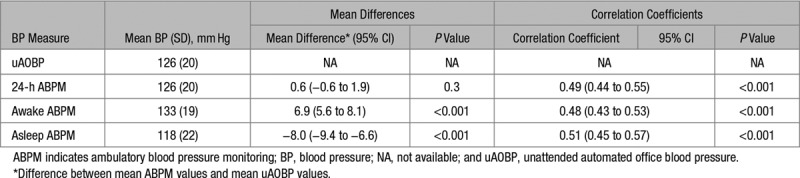
Differences and Correlation Between Systolic uAOBP and ABPM Values

### Validity Measures

**Figure 3.**
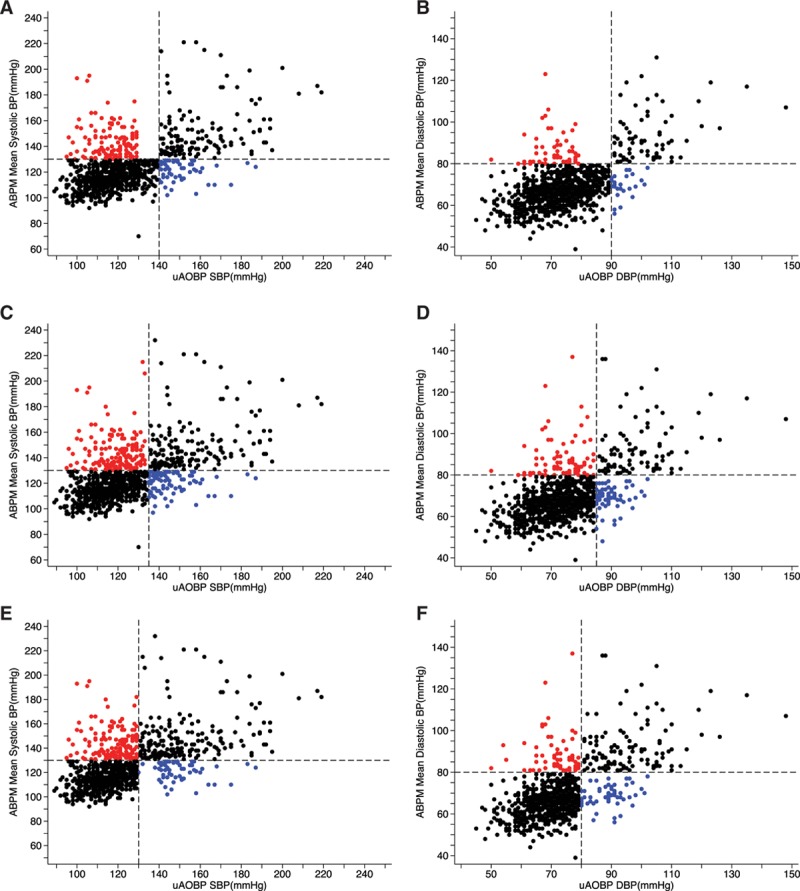
Scatter plot of ambulatory blood pressure monitoring (ABPM) vs unattended automated office blood pressure (uAOBP) values in study participants. Vertical dashed lines indicate the different systolic blood pressure (SBP)/diastolic blood pressure (DBP) cutoffs for screen positivity on uAOBP. **A**, SBP ≥140; (**B**) DBP ≥90; (**C**) SBP ≥135; (**D**) DBP ≥85; (**E**) SBP ≥130; (**F**) DBP ≥80. Horizontal dashed lines indicate cutoff for confirmed hypertension on ABPM. Data points in black indicate participants whose hypertensive status was correctly classified by uAOBP (ie, true positives and true negatives). Data points in red indicate false negatives (masked hypertension). Data points in blue indicate false positives (white coat hypertension). BP indicates blood pressure.

The proportions of participants in the different diagnostic categories according to the 3 uAOBP thresholds are displayed in Figure 3 and Table S3. The proportions of participants who screened positive on uAOBP at the ≥130/80-, ≥135/85-, and ≥140/90-mm Hg cutoffs were 45%, 32%, and 22%, respectively. The number of participants who met the definition for hypertension based on 24-hour ABPM (confirmed hypertensives) was 347 (35%).

Overall accuracy of uAOBP in determining hypertensive status as measured using area under the receiver operating curves did not differ much when using the 3 different cutoffs for defining screen positives (Tables 3 and 4). The sensitivity of uAOBP displayed an inverse association (*P*<0.001) with the cutoff selected, progressively decreasing from 67% (95% CI, 62–72) when using a cutoff of ≥130/80 mm Hg to 55% (95% CI, 49–60) at a cutoff of 135/85 mm Hg to 44% (95% CI, 39–49) at a cutoff of ≥140/90 mm Hg. Sensitivity measures were not significantly different when comparing uAOBP to 24-hour ABPM measurements than when using awake (daytime) ABPM measurements as the reference standard.

**Table 3.**
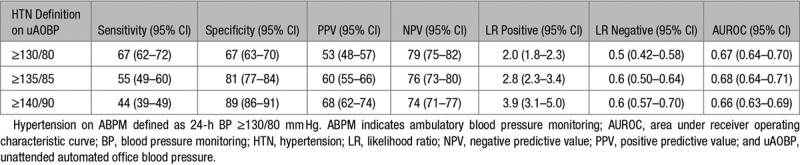
Validity of uAOBP in Diagnosing Hypertension using 24-h ABPM as Reference Standard

**Table 4.**
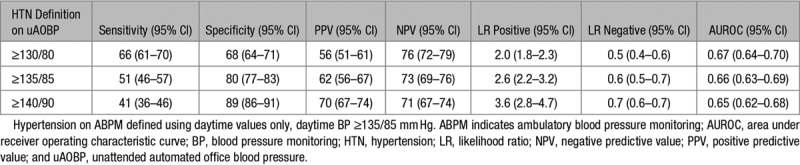
Validity of uAOBP in Diagnosing Hypertension using Daytime (Awake) ABPM as Reference Standard

### Stratified Analyses

Validity measures were not appreciably different by age group (Tables S4 through S6), sex (Tables S7 through S9), or site (Tables S10 through S12). Overall performance of uAOBP as determined by area under the receiver operating curves was not significantly different by BMI category, but sensitivity improved with increasing BMI (*P*<0.001; Tables S13 through S15). For example, at a cutoff of ≥130/80 mm Hg, for defining screen positives, sensitivity of uAOBP in obese (BMI, >30 kg/m^2^) individuals was 86% (95% CI, 74–94) compared with 59% (95% CI, 52–65) among normal-weight individuals (Table S13). In multivariable linear regression analysis, none of the factors tested was found to independently influence the difference in SBP, although sex appeared to influence diastolic differences (Table S16).

### Sensitivity Analyses

The mean 24-hour SBP in the 65 (6%) participants who reported having taken antihypertensive medication in the previous 2 weeks was 144±24 mm Hg, and only 28% of these participants had their BP under control. Inclusion of data from these participants did not materially change the results (Table S17). Inclusion of data from participants whose ABPM measurements did not meet quality control criteria did not materially change the results (Table S18).

## Discussion

In this study, we determined the accuracy of uAOBP measurement in predicting hypertensive status when screening participants drawn from the general population in Kenya. We found modest accuracy of uAOBP, with significant proportions of misclassified individuals. This was despite mean BPs on uAOBP being essentially the same as 24-hour ABPM values. Our findings suggest that additional assessment of individuals undergoing screening for hypertension is needed, which at present requires out-of-office measurements such as ABPM or home (self-measured) BP monitoring.

Our findings are at variance with most previous studies that had compared uAOBP to ABPM, which were summarized in 2 recent systematic reviews.^38,39^ Explanations for the difference include the fact that we studied a population that differed in ethnicity, age structure, BMI, and level of cardiovascular risk compared with the previous studies where uAOBP was compared with ABPM. These individuals were older, had a higher cardiovascular risk profile, and the majority were already on treatment for hypertension. These differences illustrate the problem of spectrum bias when assessing studies of diagnostic accuracy.^40^ The data we present are, therefore, more applicable to populations such as ours, which was more representative of populations undergoing screening for hypertension than previous studies of uAOBP.

As expected when using a single screening tool, the overall test performance of uAOBP as measured using the area under receiver operator characteristic curves did not differ much when 3 different cutoffs were used to define screen positives. Sensitivity of uAOBP increased significantly from 48% at a cutoff of 140/90 mm Hg to 71% at 130/80 mm Hg. This increased sensitivity without a significant change to overall accuracy provides indirect support for using 130/80 mm Hg as a cutoff for defining screen positives for hypertension in our population when using uAOBP. The increased sensitivity for detecting true hypertension may also explain the findings of the SPRINT trial^3^ where a lower treatment target was associated with an improvement in outcomes, but a report on the relationship between ABPM and uAOBP in SPRINT did not report diagnostic performance of uAOBP in the trial.^41^ Although lowering the threshold for screen positivity comes at a cost of an increase in false positives (white coat hypertensives), at a population level, this could be argued to be an acceptable tradeoff for the following reasons: first, the burden on undiagnosed hypertension in Sub-Saharan Africa is large,^19^ and, therefore, a strategy that minimizes the number of false negatives (masked hypertensives), which is common among individuals of African descent and has much worse clinical consequences than either sustained or white coat hypertension,^23^ should lead to significant population-wide benefits. Second, the current trend toward lower BP treatment targets^35^ and demonstration that white coat hypertension is not completely benign^23^ means that it might actually be beneficial to consider additional evaluation or treatment for such individuals. The 2017 American Heart Association/American College of Cardiology Hypertension Clinical Practice Guidelines lowered the cutoff for defining hypertension in American adults to an office BP ≥130/80 mm Hg—a decision that was heavily influenced by the results of SPRINT.^3^ However, the guidelines did not specifically recommend uAOBP, the procedure used in SPRINT as the preferred method to use in measuring office BP, instead stated that the diagnosis of hypertension should be “based on an average of ≥2 careful readings obtained on ≥2 occasions.” The results of our study suggest that a cutoff of ≥130/80 mm Hg when using uAOBP to measure BP may be appropriate in settings such as ours although this needs to be confirmed using data from cardiovascular disease outcome studies.

A significant strength of this study was that we applied uAOBP to the purpose (screening) that it will be used for on most individuals in both high- and low-income settings. In virtually all settings, there is a care cascade with a progressive drop in numbers starting from those undergoing screening to those initiating treatment and those getting their BP under control. We also studied a population that is more reflective of that undergoing screening in Kenya and other settings.^19,20,42^ Our sample size was significantly larger than most of the previously conducted studies^18,38,39^ enabling us to obtain more precise estimates of validity. These validity measures, which were robust to several sensitivity analyses, will be useful in estimating the relative cost-effectiveness of different strategies for diagnosing hypertension, which is important for policy makers who have to make decisions in the face of competing health priorities in Africa.

The main limitation of this study was the absence of locally derived cardiovascular disease outcome-based data for defining the cutoffs for both uAOBP and ABPM values, which led us to use internationally derived definitions. uAOBP is a relatively new method of BP measurement, and few studies^43^ worldwide have been conducted to determine cardiovascular disease outcomes (and, therefore, set thresholds) in relation to uAOBP measurements.^8^ Regarding ABPM, we used widely accepted definitions that have been derived from multiethnic cohorts.^4,8^ It is, however, possible that the threshold values are different in our setting, as suggested by an analysis from the Jackson Heart Study that proposed a higher threshold for defining hypertension using ABPM among blacks.^44^ Few if any cohort or randomized studies of hypertension in relation to outcomes have been conducted in Africa,^45^ and until such data are available, there will be continued reliance on externally derived guidelines. The differences we have observed in the relationship between uAOBP and ABPM in our setting compared with that described in high-income settings emphasize the need for additional studies. We also intend to update the analyses presented here once sufficient follow-up time has accrued to determine outcomes in relation to the BP measurements that were taken. Even if these subsequent data support the thresholds that we used, the logistical challenges of implementing out-of-office measurements as suggested by this study’s findings will have to be considered. Algorithms for determining which individuals to refer for out-of-office measurements may help in reducing the amount of resources used in conducting these measurements.^46^

Another limitation is that we excluded some data because of poor-quality ABPM measurements in a small proportion of participants. Other than slightly reducing the precision of our estimates, no other effects of this loss of data are likely have occurred because >80% of participants who underwent uAOBP also had acceptable ABPM data, thus minimizing the risk of partial or differential verification bias.^47^ Also, because of small numbers on stratifying by age, we were unable to determine whether the relationship between uAOBP and ABPM differs with age as has been demonstrated for the relationship between conventional office BP and ABPM.^48^ Although we observed significant differences when stratifying by BMI, the numbers of obese and overweight individuals were small, and these findings need to be confirmed in larger studies. We were not powered to detect differences by study site, although stratified analyses suggested that if present, these were not large in magnitude. We were also not powered to analyze predictors of lack of agreement between uAOBP and ABPM. Staff were not blinded to the results of the measurements but because uAOBP measurements were performed with the participants alone in the room and the majority of ABPM measurements for individual participants over the 24-hour period were outside the clinic, it is unlikely that this introduced any bias.

In conclusion, in this study conducted among the general population at 3 sites in Kenya, we found modest levels of accuracy of uAOBP in screening for hypertension, suggesting that out-of-office BP measurements are needed in confirming hypertensive status of individuals. Additional studies based on cardiovascular outcomes that also incorporate implementation science are needed to better define screening strategies for hypertension.

## Perspectives

This study in which we found modest levels of diagnostic accuracy of uAOBP measurement when screening for hypertension illustrates the difficulties in finding an accurate and broadly applicable method for measuring BP in the office. Additional studies in different settings and population groups are required.

## Acknowledgments

We would like to thank all participants who participated in the study together with their families, as well as the staff at the KEMRI Wellcome Trust Research Programme. We thank Dr Kimani Gachuhi, Dr Joseph Mwatha, and other members of the NAHENDA study group (National Hypertension and Diabetes Survey) for help in the early stages of planning the study. This article is published with the approval of the Director, Kenya Medical Research Institute.

## Sources of Funding

Funds for this study were provided by an internal research grant by KEMRI to A.O. Etyang (grant No. KEMRI/GRG/15/09). A.O. Etyang was additionally funded by the Wellcome Trust (fellowship No. 103951/Z/14/Z). The Funders played no role in the preparation of this article.

## Disclosures

None.

## Supplementary Material

## Novelty and Significance

### What Is New?

- We determined the accuracy of unattended automated office blood pressure measurement when screening individuals from the general population for hypertension in Kenya.

### What Is Relevant?

- Blood pressure measurement is a critical part of cardiovascular risk assessment. There is increasing adoption of unattended automated office blood pressure measurement worldwide, but its performance when screening for hypertension in the general population has not been assessed.

### Summary

We found modest accuracy of unattended automated office blood pressure measurement when screening for hypertension among individuals drawn from the general population in Kenya. This suggests that out-of-office measurements are needed to confirm a diagnosis of hypertension.
