# Supplementary material for: Diagnostic Accuracy of Unattended Automated Office Blood Pressure Measurement in Screening for Hypertension in Kenya
Source: Hypertension. 2019 Oct 28;74(6):1490–8. doi: 10.1161/HYPERTENSIONAHA.119.13574 (PMC7069390; doi:10.1161/HYPERTENSIONAHA.119.13574)
Supplement: Supplementary file 1 [file hyp-74-1490-s001.docx]

**ONLINE SUPPLEMENT**

Diagnostic Accuracy of Unattended Automated Office Blood Pressure Measurement in Screening for Hypertension in Kenya

**Authors**

Anthony O. Etyang, MBChB, MMed, Msc, PhD^1^

Antipa Sigilai, BSc^1^

Emily Odipo, BSc^1^

Robinson Oyando, BSc^2^

Gerald Ong’ayo, MBChB^1^

Lawrence Muthami, PhD^3^

Kenneth Munge, MBChB, MPH^2^

Fredrick Kirui, MBChB, MMed, MPH^4^

Jane Mbui, MBChB, MSc, MMed^4^

Zipporah Bukania, PhD^3^

Judy Mwai, MSc^3^

Andrew Obala, PhD^5^

Edwine Barasa, PhD^2^

**Author Affiliations**

^1^Centre for Geographic Medicine Research, Kenya Medical Research Institute, Kilifi Kenya

^2^ Health Economics Research Unit, KEMRI-Wellcome Trust Research Programme

^3^Centre for Public Health Research, Kenya Medical Research Institute, Nairobi, Kenya

^4^Centre for Clinical Research, Kenya Medical Research Institute, Nairobi, Kenya

^5^Moi University, Eldoret, Kenya

**Table S1: Characteristics of study participants at each site**

| Characteristics |  | Kilifi  N=404 | | | Kirinyaga  N=246 | |  | Webuye  N=339 | |
| --- | --- | --- | --- | --- | --- | --- | --- | --- | --- |
|  |  | N | *(%)* |  | N | *(%)* |  | N | *(%)* |
| Female |  | 223 | *(55)* |  | 166 | *(67)* |  | 187 | *(56)* |
| Smoker |  | 51 | *(13)* |  | 21 | *(9)* |  | 17 | *(5)* |
| Diabetes mellitus |  | 10 | *(2)* |  | 4 | *(2)* |  | 4 | *(1)* |
| Previously diagnosed hypertensive^1^ |  | 13 | *(6)* |  | 20 | *(9)* |  | 19 | *(12)* |
|  |  | mean | *(SD)* |  | mean | *(SD)* |  | Mean | *(SD)* |
| Age, years |  | 40 | *(17)* |  | 43 | *(14)* |  | 44 | *(17)* |
| BMI kg/m^2^ |  | 21 | *(4)* |  | 26 | *(6)* |  | 22 | *(8)* |
| Blood pressure, mm Hg  uAOBP |  |  |  |  |  |  |  |  |  |
| Systolic |  | 123 | *(20)* |  | 125 | *(19)* |  | 129 | *(20)* |
| Diastolic |  | 73 | *(13)* |  | 78 | *(12)* |  | 79 | *(12)* |
| ABPM |  |  |  |  |  |  |  |  |  |
| 24-hour systolic |  | 125 | *(19)* |  | 129 | *(21)* |  | 126 | *(19)* |
| 24-hour diastolic |  | 70 | *(12)* |  | 73 | *(14)* |  | 71 | *(13)* |

BMI: Body mass index; SD: standard deviation; ABPM: ambulatory blood pressure monitoring

^1^Answered “yes” to the question: Has a doctor or healthcare worker previously told you that you have high blood pressure?

Diagnosis of Diabetes Mellitus was based on fasting blood sugar result ≥7.1 mmol/L or previous diagnosis of Diabetes Mellitus

**Table S2: Differences and correlations between Diastolic uAOBP and ABPM values**

|  | Mean differences | | | |  | Correlation coefficients | | |
| --- | --- | --- | --- | --- | --- | --- | --- | --- |
| BP measure | Mean BP (mm Hg) | *(SD)* | Mean difference^±^, (95% CI) | p-value |  | Correlation coefficient | (95% CI) | p-value |
| uAOBP | 76 | *(13)* | N/A | N/A |  | N/A | N/A | N/A |
| 24hr ABPM | 71 | *(13)* | –5.2 (–5.9, –4.5) | <0.001 |  | 0.54 | (0.48, 0.59) | <0.001 |
| Awake ABPM | 76 | *(13)* | –0.6 (–1.4, 0.2) | 0.129 |  | 0.55 | (0.49, 0.60) | <0.001 |
| Asleep ABPM | 65 | *(13)* | –11 (–12, –10.6) | <0.001 |  | 0.51 | (0.45, 0.56) | <0.001 |

^±^ Difference between mean ABPM values and mean uAOBP values

uAOBP: unattended Automated Office Blood Pressure

ABPM: Ambulatory blood pressure monitoring

DBP: Diastolic blood pressure

**Table S3: Proportions of white coat hypertensives and masked hypertensives at different uAOBP thresholds**

| Diagnostic Category | Screen positive definition on uAOBP | | | | | | | |
| --- | --- | --- | --- | --- | --- | --- | --- | --- |
|  | ≥ 130/80 mm Hg | |  | ≥ 135/ 85 mm Hg | |  | ≥ 140/ 90 mm Hg | |
|  | N | (%) |  | N | (%) |  | N | (%) |
| Sustained normotensives | 424 | (43) |  | 511 | (52) |  | 564 | (57) |
| Sustained hypertensives | 233 | (24) |  | 189 | (19) |  | 152 | (15) |
| White coat hypertensives | 211 | (21) |  | 124 | (13) |  | 71 | (7) |
| Masked hypertensives | 114 | (11) |  | 158 | (16) |  | 195 | (20) |

**Table S4: Age stratified validity measures (Screen positive= uAOBP ≥ 130/80 mm Hg)**

| Age category | N | Proportion with HTN  (95% CI) | Sensitivity | Specificity | PPV | NPV | LR positive | LR negative | AUROC  (95% CI) |
| --- | --- | --- | --- | --- | --- | --- | --- | --- | --- |
| 18-29 | 281 | 26(21, 31) | 44(33, 57) | 79(72, 84) | 42(30, 53) | 80(74, 86) | 2.1(1.4, 3.0) | 0.7(0.6, 0.9) | 0.62(0.55, 0.68) |
| 30-39 | 209 | 25(20, 32) | 60(46, 74) | 76(69, 83) | 46(34, 59) | 85(78, 91) | 2.6(1.8, 3.6) | 0.5(0.4, 0.7) | 0.68(0.61, 0.76) |
| 40-49 | 199 | 41(34, 48) | 67(55, 77) | 59(50, 68) | 53(43, 63) | 72(62, 81) | 1.6(1.3, 2.1) | 0.6(0.4, 0.8) | 0.63(0.56, 0.70) |
| 50-59 | 128 | 43(34, 52) | 91(80, 97) | 53(41, 65) | 60(48, 70) | 89(75, 96) | 2.0(1.5, 2.5) | 0.2(0.1, 0.4) | 0.72(0.65, 0.79) |
| 60-69 | 102 | 47(37, 57) | 73(58, 85) | 33(21, 48) | 49(37, 61) | 58(39, 76) | 1.1(0.8,1.4) | 0.8(0.5, 1.5) | 0.53(0.44, 0.62) |
| 70+ | 63 | 60(47, 72) | 79(63, 90) | 56(35, 76) | 73(57, 86) | 64(41, 83) | 1.8(1.1, 2.9) | 0.4(0.2, 0.8) | 0.68(0.56, 0.79) |

PPV: positive predictive value; NPV; negative predictive value; LR: likelihood ratio; AUROC: area under receiver operating characteristic curve

**Table S5: Age stratified validity measures (Screen positive= uAOBP ≥ 135/85 mm Hg)**

| Age category | N | Proportion with HTN  (95% CI) | Sensitivity | Specificity | PPV | NPV | LR positive | LR negative | AUROC  (95% CI) |
| --- | --- | --- | --- | --- | --- | --- | --- | --- | --- |
| 18-29 | 281 | 26(21, 31) | 31(20, 43) | 92(87, 95) | 56(40, 72) | 79(74, 84) | 3.8(2.1, 6.7) | 0.8(0.6, 0.9) | 0.61(0.56, 0.67) |
| 30-39 | 209 | 25(20, 32) | 42(28, 56) | 87(80, 92) | 51(36, 67) | 81(74, 87) | 3.1(1.9, 5.1) | 0.7(0.5, 0.9) | 0.64(0.57, 0.71) |
| 40-49 | 199 | 41(34, 48) | 52(41, 643) | 80(71, 87) | 64(51, 75) | 71(62, 78) | 2.6(1.7, 3.9) | 0.6(0.5, 0.8) | 0.66(0.59, 0.72) |
| 50-59 | 128 | 43(34, 52) | 84(71, 92) | 67(55, 78) | 66(53, 77) | 85(73, 93) | 2.5(1.8, 3.6) | 0.2(0.1, 0.5) | 0.75(0.68, 0.83) |
| 60-69 | 102 | 47(37, 57) | 67(52, 80) | 48(34, 62) | 53(40, 66) | 52(46, 76) | 1.3(0.9, 1.8) | 0.7(0.4, 1.1) | 0.57(0.48, 0.67) |
| 70+ | 63 | 60(47, 72) | 66(49, 80) | 60(39, 79) | 71(54, 85) | 54(34, 73) | 1.6(1.0, 2.8) | 0.6(0.3, 1.0) | 0.63(0.51, 0.75) |

PPV: positive predictive value; NPV; negative predictive value; LR: likelihood ratio; AUROC: area under receiver operating characteristic curve

**Table S6: Age stratified validity measures (Screen positive= uAOBP ≥ 140/90 mm Hg)**

| Age category | N | Proportion with HTN  (95% CI) | Sensitivity | Specificity | PPV | NPV | LR positive | LR negative | AUROC  (95% CI) |
| --- | --- | --- | --- | --- | --- | --- | --- | --- | --- |
| 18-29 | 281 | 26(21, 31) | 15(8, 26) | 96(93, 98) | 58(34, 80) | 77(71, 82) | 4.0(1.7, 9.5) | 0.9(0.8, 1.0) | 0.56(0.51, 0.60) |
| 30-39 | 209 | 25(20, 32) | 30(18, 44) | 93(88, 96) | 59(39, 78) | 80(73, 85) | 4.3(2.1, 8.6) | 0.8(0.6, 0.9) | 0.62(0.55, 0.68) |
| 40-49 | 199 | 41(34, 48) | 41(30, 52) | 88(81, 93) | 70(55, 83) | 68(60, 76) | 3.4(2.0, 6.0) | 0.7(0.6, 0.8) | 0.64(0.58, 0.71) |
| 50-59 | 128 | 43(34, 52) | 73(59, 84) | 84(73, 91) | 77(63, 88) | 80(70, 89) | 4.4(2.6, 7.6) | 0.3(0.2, 0.5) | 0.78(0.71, 0.86) |
| 60-69 | 102 | 47(37, 57) | 63(47, 76) | 65(51, 77) | 61(46, 74) | 66(52, 79) | 1.8(1.2, 2.7) | 0.6(0.4, 0.9) | 0.64(0.54, 0.73) |
| 70+ | 63 | 60(47, 72) | 58(41, 74) | 72(51, 88) | 76(57, 90) | 53(35, 70) | 2.1(1.0, 4.1) | 0.6(0.4, 0.9) | 0.65(0.53, 0.77) |

PPV: positive predictive value; NPV; negative predictive value; LR: likelihood ratio; AUROC: area under receiver operating characteristic curve

**Table S7: Validity measures by sex (Screen positive= uAOBP ≥ 130/80 mm Hg)**

| Sex | N | True HTN  (95% CI) | Sensitivity, (95% CI) | Specificity, (95% CI) | PPV,  (95% CI) | NPV,  (95% CI) | LR positive, (95% CI) | LR negative, (95% CI) | AUROC  (95% CI) |
| --- | --- | --- | --- | --- | --- | --- | --- | --- | --- |
| Women | 576 | 33(29,37) | 73(66, 79) | 69(64,73) | 53(47, 59) | 84(79, 88) | 2.3(1.9, 2.7) | 0.4(0.32, 0.51) | 0.71(0.67, 0.74) |
| Men | 406 | 39(34,44) | 61(53, 68) | 64(58,70) | 52(44, 59) | 72(66, 78) | 1.7(1.4, 2.1) | 0.6(0.49, 0.76) | 0.62(0.58, 0.67) |

**Table S8: Validity measures by sex (Screen positive= uAOBP ≥ 135/85 mm Hg)**

| Sex | N | True HTN  (95% CI) | Sensitivity, (95% CI) | Specificity, (95% CI) | PPV,  (95% CI) | NPV,  (95% CI) | LR positive, (95% CI) | LR negative, (95% CI) | AUROC  (95% CI) |
| --- | --- | --- | --- | --- | --- | --- | --- | --- | --- |
| Women | 576 | 33(29,37) | 57(50, 64) | 81(77, 85) | 60(52, 67) | 80(75, 83) | 3.0(2.4, 3.9) | 0.5(0.45, 0.63) | 0.69(0.65, 0.73) |
| Men | 406 | 39(34,44) | 51(43, 59) | 79(74, 84) | 61(53, 70) | 72(66, 77) | 2.5(1.9, 3.3) | 0.6(0.52, 0.73) | 0.65(0.61, 0.70) |

**Table S9: Validity measures by sex (Screen positive= uAOBP ≥ 140/90 mm Hg)**

| Sex | N | True HTN  (95% CI) | Sensitivity, (95% CI) | Specificity, (95% CI) | PPV,  (95% CI) | NPV,  (95% CI) | LR positive, (95% CI) | LR negative, (95% CI) | AUROC  (95% CI) |
| --- | --- | --- | --- | --- | --- | --- | --- | --- | --- |
| Women | 576 | 33(29,37) | 50(42, 57) | 90(86, 93) | 70(62, 78) | 79(74, 82) | 4.8(3.5, 6.7) | 0.6(0.49, 0.65) | 0.70(0.66, 0.74) |
| Men | 406 | 39(34,44) | 37(29, 45) | 88(83, 91) | 65(54, 74) | 69(63, 74) | 2.9(2.0, 4.3) | 0.7(0.64, 0.82) | 0.62(0.58, 0.66) |

PPV: positive predictive value; NPV; negative predictive value; LR: likelihood ratio; AUROC: area under receiver operating characteristic curve

**Table S10: Validity measures by site (Screen positive= uAOBP ≥ 130/80 mm Hg)**

| Site | N | True HTN  (95% CI) | Sensitivity, (95% CI) | Specificity, (95% CI) | PPV,  (95% CI) | NPV,  (95% CI) | LR positive, (95% CI) | LR negative, (95% CI) | AUROC  (95% CI) |
| --- | --- | --- | --- | --- | --- | --- | --- | --- | --- |
| Kilifi | 404 | 32(28,37) | 62(53, 71) | 78(72,83) | 57(49, 65) | 81(76, 86) | 2.8(2.2, 3.6) | 0.5(0.39, 0.61) | 0.70(0.65, 0.75) |
| Webuye | 332 | 35(30,40) | 72(63, 80) | 56(49,63) | 47(39, 55) | 79(72, 85) | 1.7(1.4, 2.0) | 0.5(0.36, 0.68) | 0.64(0.59, 0.70) |
| Kirinyaga | 246 | 41(35,48) | 67(57, 76) | 62(54,70) | 55(46, 81) | 73(64, 81) | 1.8(1.4, 2.3) | 0.5(0.39, 0.71) | 0.65(0.59, 0.71) |

**Table S11: Validity measures by site (Screen positive= uAOBP ≥ 135/85 mm Hg)**

| Site | N | True HTN  (95% CI) | Sensitivity, (95% CI) | Specificity, (95% CI) | PPV,  (95% CI) | NPV,  (95% CI) | LR positive, (95% CI) | LR negative, (95% CI) | AUROC  (95% CI) |
| --- | --- | --- | --- | --- | --- | --- | --- | --- | --- |
| Kilifi | 404 | 32(28,37) | 54(45, 63) | 88(84, 92) | 68(58, 77) | 80(75, 84) | 4.5(3.1, 6.4) | 0.5(0.43, 0.64) | 0.71(0.66, 0.76) |
| Webuye | 332 | 35(30,40) | 55(46, 64) | 75(68, 80) | 54(44, 63) | 76(69, 81) | 2.2(1.6, 2.9) | 0.6(0.48, 0.75) | 0.65(0.60, 0.70) |
| Kirinyaga | 246 | 41(35,48) | 55(44, 64) | 75(67, 82) | 60(50, 71) | 70(63, 78) | 2.2(1.6, 3.1) | 0.6(0.48, 0.77) | 0.65(0.59, 0.71) |

**Table S12: Validity measures by site (Screen positive= uAOBP ≥ 140/90 mm Hg)**

| Site | N | True HTN  (95% CI) | Sensitivity, (95% CI) | Specificity, (95% CI) | PPV,  (95% CI) | NPV,  (95% CI) | LR positive, (95% CI) | LR negative, (95% CI) | AUROC  (95% CI) |
| --- | --- | --- | --- | --- | --- | --- | --- | --- | --- |
| Kilifi | 404 | 32(28,37) | 43(34, 52) | 93(89, 96) | 75(63, 84) | 78(73, 82) | 6.2(3.9, 10) | 0.6(0.53, 0.71) | 0.68(0.64, 0.73) |
| Webuye | 332 | 35(30,40) | 47(37, 56) | 86(80, 90) | 64(52, 74) | 75(69, 80) | 3.2(2.2, 4.7) | 0.6(0.52, 0.75) | 0.66(0.61, 0.71) |
| Kirinyaga | 246 | 41(35,48) | 42(32, 52) | 86(79, 91) | 67(54, 78) | 68(61, 75) | 2.9(1.8, 4.5) | 0.7(0.57, 0.82) | 0.64(0.58, 0.69) |

True HTN: crude prevalence of hypertension as determined using 24-hour ABPM; PPV: positive predictive value; NPV; negative predictive value; LR: likelihood ratio; AUROC: area under receiver operating characteristic curve

**Table S13: Validity measures by BMI category (Screen positive= uAOBP ≥ 130/80 mm Hg)**

| BMI(Kg/m^2^) | N | True HTN  (95% CI) | Sensitivity, (95% CI) | Specificity, (95% CI) | PPV,  (95% CI) | NPV,  (95% CI) | LR positive, (95% CI) | LR negative, (95% CI) | AUROC  (95% CI) |
| --- | --- | --- | --- | --- | --- | --- | --- | --- | --- |
| <25 | 724 | 32(29,36) | 59(52, 65) | 70(66,74) | 49(43, 55) | 78(74, 82) | 2.0(1.7, 2.4) | 0.6(0.50, 0.69) | 0.65(0.61, 0.68) |
| 25-29 | 160 | 34(26,42) | 85(73, 93) | 57(47,66) | 50(39, 61) | 88(78, 95) | 2.0(1.5, 2.5) | 0.3(0.14, 0.51) | 0.71(0.64, 0.78) |
| >30 | 93 | 61(51,71) | 86(74, 94) | 50(33,67) | 73(61,83) | 69(48, 86) | 1.7(1.2, 2.4) | 0.3(0.14, 0.58) | 0.68(0.59, 0.77) |

**Table S14: Validity measures by BMI category (Screen positive= uAOBP ≥ 135/85 mm Hg)**

| BMI(Kg/m^2^) | N | True HTN  (95% CI) | Sensitivity, (95% CI) | Specificity, (95% CI) | PPV,  (95% CI) | NPV,  (95% CI) | LR positive, (95% CI) | LR negative, (95% CI) | AUROC  (95% CI) |
| --- | --- | --- | --- | --- | --- | --- | --- | --- | --- |
| <25 | 724 | 32(29,36) | 46(40, 53) | 84(81, 87) | 58(51, 65) | 76(73, 80) | 2.9(2.3, 3.7) | 0.6(0.57, 0.73) | 0.65(0.61, 0.69) |
| 25-29 | 160 | 34(26,42) | 74(60, 85) | 72(62, 80) | 57(45, 69) | 84(75, 91) | 2.6(1.9, 3.7) | 0.4(0.22, 0.58) | 0.73(0.66, 0.80) |
| >30 | 93 | 61(51,71) | 72(59, 83) | 61(44, 77) | 75(61, 85) | 58(41, 74) | 1.9(1.2, 2.9) | 0.5(0.28, 0.75) | 0.67(0.57, 0.77) |

**Table S15: Validity measures by BMI category (Screen positive= uAOBP ≥ 140/90 mm Hg)**

| BMI(Kg/m^2^) | N | True HTN  (95% CI) | Sensitivity, (95% CI) | Specificity, (95% CI) | PPV,  (95% CI) | NPV,  (95% CI) | LR positive, (95% CI) | LR negative, (95% CI) | AUROC  (95% CI) |
| --- | --- | --- | --- | --- | --- | --- | --- | --- | --- |
| <25 | 724 | 32(29,36) | 35(29,42) | 92(89, 94) | 67(58, 75) | 75(71, 78) | 4.2(3.0, 5.9) | 0.7(0.64, 0.78) | 0.64(0.60, 0.67) |
| 25-29 | 160 | 34(26,42) | 59(45, 72) | 81(72, 88) | 62(47, 75) | 80(71, 87) | 3.1(2.0, 4.9) | 0.5(0.36, 0.70) | 0.70(0.63, 0.78) |
| >30 | 93 | 61(51,71) | 65(51, 77) | 75(58, 88) | 80(66, 91) | 57(42, 72) | 2.6(1.4, 4.7) | 0.5(0.31, 0.70) | 0.70(0.60, 0.80) |

True HTN: crude prevalence of hypertension as determined using 24-hour ABPM; PPV: positive predictive value; NPV; negative predictive value; LR: likelihood ratio; AUROC: area under receiver operating characteristic curve

**Table S16: Independent factors associated with differences between uAOBP and 24-hour ABPM values in fully adjusted multiple regression models**

| Predictor variable | Systolic BP difference | |  | Diastolic BP difference | |
| --- | --- | --- | --- | --- | --- |
|  | Coefficient | *(95% CI)* |  | Coefficient | (95% CI) |
| Age (per 1 year increment) | .0004 | *(–.0002, .001)* |  | .0001 | *(–.001, .001)* |
| Sex (reference=female) | .001 | *(–.02, .02)* |  | –.03 | *(–.05, –.003)* |
| BMI category (normal, overweight, obese) | .01 | *(–.01, .01)* |  | .01 | *(–.01, .03)* |
| Study site | .0001 | *(–.01, .01)* |  | .006 | *(–.05, .04)* |
| Presence of diabetes mellitus | .02 | *(–.02, .04)* |  | .03 | *(–.05, .03)* |
| Smoking status | .01 | *(–.02, .04)* |  | –.01 | *(–.05, .04)* |

Coefficients reflect the increase in the BP difference per change in the variable unit. BMI was categorized as normal (<25 kg/m^2^), overweight (25-29.9 kg/m^2^) and obese (≥30 kg/m^2^)

**Table S17: Validity measures including 65 participants who were taking anti-hypertensive medications (N=1,047)**

| HTN definition on uAOBP | Sensitivity,  (95% CI) | Specificity,  (95% CI) | PPV,  (95% CI) | NPV,  (95% CI) | LR positive, (95% CI) | LR negative, (95% CI) | AUROC  (95% CI) |
| --- | --- | --- | --- | --- | --- | --- | --- |
| ≥ 130/80 | 71(66, 75) | 66(62,70) | 56(51, 60) | 79(75, 82) | 2.1(1.8, 2.3) | 0.4(0.38, 0.52) | 0.68(0.65, 0.71) |
| ≥ 135/85 | 58(53, 63) | 80(76, 83) | 63(58, 68) | 76(72, 79) | 2.8(2.4, 3.4) | 0.5(0.47, 0.60) | 0.69(0.66, 0.71) |
| ≥ 140/90 | 48(43, 53) | 88(85, 91) | 70(65, 76) | 74(70, 77) | 3.9(3.1, 5.0) | 0.6(0.54, 0.66) | 0.68(0.65, 0.71) |

**Table S18: Validity measures including participants with poor quality ABPM data (N=1,291)**

| HTN definition on uAOBP | Sensitivity, (95% CI) | Specificity, (95% CI) | PPV,  (95% CI) | NPV,  (95% CI) | LR positive, (95% CI) | LR negative, (95% CI) | AUROC  (95% CI) |
| --- | --- | --- | --- | --- | --- | --- | --- |
| ≥ 130/80 | 68(64, 72) | 67(63,70) | 55(51, 59) | 78(75, 81) | 2.1(1.8, 2.3) | 0.5(0.41, 0.55) | 0.68(0.65, 0.70) |
| ≥ 135/85 | 55(50, 60) | 80(77, 83) | 62(57, 66) | 75(72, 78) | 2.7(2.3, 3.2) | 0.6(0.51, 0.63) | 0.68(0.65, 0.70) |
| ≥ 140/90 | 45(41, 50) | 88(86, 90) | 69(63, 74) | 73(70, 76) | 3.7(3.0, 4.6) | 0.6(0.58, 0.68) | 0.67(0.64, 0.69) |

229 participants had poor quality ABPM data and 1047 had good quality ABPM data. ABPM data quality was defined using IDACO criteria (≥10 daytime readings and ≥ 5 nighttime readings)

**Table S19: Validity measures including participants with non-dipping BP pattern on ABPM data (N=184)**

| HTN definition on uAOBP | Sensitivity, (95% CI) | Specificity, (95% CI) | PPV,  (95% CI) | NPV,  (95% CI) | LR positive, (95% CI) | LR negative, (95% CI) | AUROC  (95% CI) |
| --- | --- | --- | --- | --- | --- | --- | --- |
| ≥ 130/80 | 70(60, 79) | 64(53,74) | 69(59, 78) | 66(54, 76) | 2.0(1.4, 2.7) | 0.5(0.33, 0.65) | 0.67(0.60, 0.74) |
| ≥ 135/85 | 57(47, 67) | 77(66, 85) | 74(62, 83) | 61(51, 70) | 2.5(1.6, 3.7) | 0.6(0.43, 0.72) | 0.67(0.60, 0.74) |
| ≥ 140/90 | 45(35, 55) | 87(78, 93) | 80(67, 90) | 58(49, 67) | 3.5(1.9, 6.4) | 0.6(0.52, 0.77) | 0.66(0.60, 0.72) |

184 participants had non-dipping 24-hour BP pattern, defined as nighttime BP higher than daytime BP

**Figure S1. Study locations**

Kilifi is located along the Indian Ocean coast, Kirinyaga is in the central highlands and Webuye is in western Kenya. All three study sites comprise predominantly rural/semi-urban populations with agriculture being the main occupation.

**Figure S2. Study flowchart in Kilifi**

**Figure S3. Study flowchart in Kirinyaga**

**Figure S4. Study flowchart in Webuye**
