# Supplementary figures and images for: Diagnostic Accuracy of Unattended Automated Office Blood Pressure Measurement in Screening for Hypertension in Kenya
Source: Hypertension. 2019 Oct 28;74(6):1490–8. doi: 10.1161/HYPERTENSIONAHA.119.13574 (PMC7069390; doi:10.1161/HYPERTENSIONAHA.119.13574)

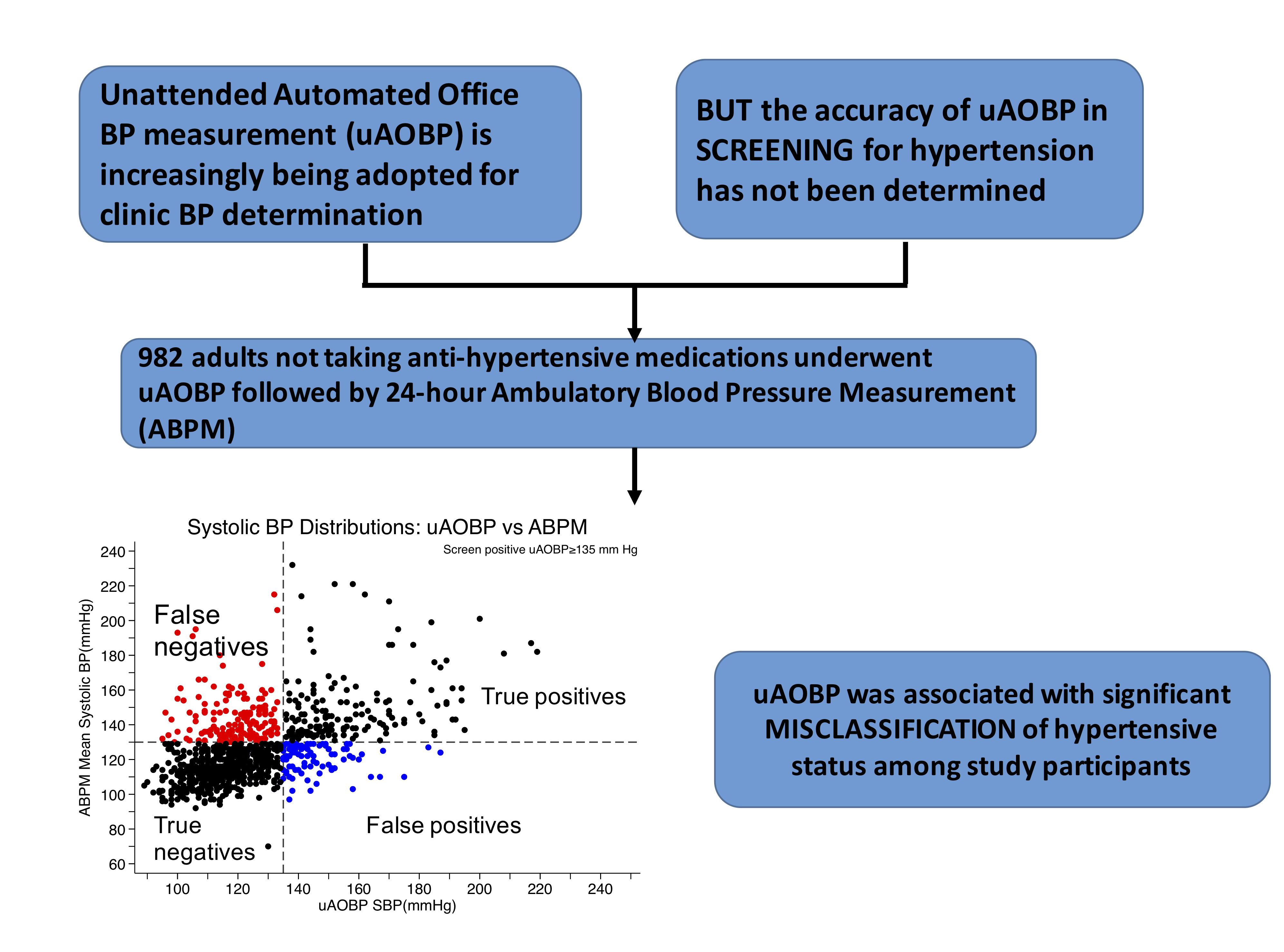

Supplement: Supplementary file 2 [file hyp-74-1490-s002.jpg]
